# Early Management of Retained Hemothorax in Blunt Head and Chest Trauma

**DOI:** 10.1007/s00268-017-4420-x

**Published:** 2018-01-05

**Authors:** Fong-Dee Huang, Wen-Bin Yeh, Sheng-Shih Chen, Yuan-Yuarn Liu, I-Yin Lu, Yi-Pin Chou, Tzu-Chin Wu

**Affiliations:** 1 Division of Trauma, Department of Emergency, Kaohsiung Veterans General Hospital, 386, Da-Chung 1st Road, Kaohsiung City, 813 Taiwan; 2 Department of Kinesiology, Health, and Leisure Studies, National University of Kaohsiung, Kaohsiung City, Taiwan; 3 Department of Cosmetic Science, College of Human Ecology, Chang Gung University of Science and Technology, Kweishan, Taoyuan, Taiwan; 4 Division of Chest Medicine, Department of Internal Medicine, Chung Shan Medical University Hospital, Taichung, Taiwan; 5 School of Medicine, Chung Shan Medical University, Taichung, Taiwan; 6 Division of Thoracic Surgery, Department of Surgery, Kaohsiung Veterans General Hospital, Kaohsiung City, Taiwan; 7 Department of Optometry, Shu-Zen Junior College of Medicine and Management, Kaohsiung, Taiwan; 8 Department of Nursing, Kaomei Junior College of Health Care and Management, Kaohsiung, Taiwan

## Abstract

**Background:**

Major blunt chest injury usually leads to the development of retained hemothorax and pneumothorax, and needs further intervention. However, since blunt chest injury may be combined with blunt head injury that typically requires patient observation for 3–4 days, other critical surgical interventions may be delayed. The purpose of this study is to analyze the outcomes of head injury patients who received early, versus delayed thoracic surgeries.

**Materials and methods:**

From May 2005 to February 2012, 61 patients with major blunt injuries to the chest and head were prospectively enrolled. These patients had an intracranial hemorrhage without indications of craniotomy. All the patients received video-assisted thoracoscopic surgery (VATS) due to retained hemothorax or pneumothorax. Patients were divided into two groups according to the time from trauma to operation, this being within 4 days for Group 1 and more than 4 days for Group 2. The clinical outcomes included hospital length of stay (LOS), intensive care unit (ICU) LOS, infection rates, and the time period of ventilator use and chest tube intubation.

**Result:**

All demographics, including age, gender, and trauma severity between the two groups showed no statistical differences. The average time from trauma to operation was 5.8 days. The ventilator usage period, the hospital and ICU length of stay were longer in Group 2 (6.77 vs. 18.55, *p* = 0.016; 20.63 vs. 35.13, *p* = 0.003; 8.97 vs. 17.65, *p* = 0.035). The rates of positive microbial cultures in pleural effusion collected during VATS were higher in Group 2 (6.7 vs. 29.0%, *p* = 0.043). The Glasgow Coma Scale score for all patients improved when patients were discharged (11.74 vs. 14.10, *p* < 0.05).

**Discussion:**

In this study, early VATS could be performed safely in brain hemorrhage patients without indication of surgical decompression. The clinical outcomes were much better in patients receiving early intervention within 4 days after trauma.

## Introduction

In Taiwan, blunt injury is the main type of trauma resulting from traffic accidents. It usually causes injuries to many different regions of the human body. Head and chest injuries usually occur together [1–3]. Managing such multiple traumas is always a great challenge; exact assessments and lifesaving procedures are mandated and should be performed rapidly. However, many surgical interventions should be delayed due to head injury because damaged brain tissue requires longer time periods of observation to maintain adequate oxygenation and perfusion [1, 2, 4].

Most major blunt chest traumas involve rib fracture with lung contusion and hemopneumothorax. About 85 % of patients could be treated with pain control or simple tube thoracostomies even with head injury [5, 6]. Some patients will develop retained pleural collections that should be managed with further surgical intervention to prevent post-traumatic complications [6–8]. Since 1990, video-assisted thoracoscopic surgery (VATS) has become a popular and acceptable method for treating retained hemothorax [5, 9–11]. Although early VATS that could improve clinical outcomes has been proofed in many studies, timing of these operations is usually influenced by associated injuries, especially the head injury. Due to the autoregulation of cerebral vascularity recovered at least 96 h after brain injuries [1, 2, 4], VATS is usually delayed until the patients’ head injuries stabilized somewhat after 4 days.

The effects of time periods to VATS in patients with both blunt head and chest trauma have rarely been studied. A hypothesis is set up in this study that early interventions may not worsen the brain condition but rather will improve final clinical outcomes. A new strategy of early management of thoracic injury with head injury is proposed. The outcomes of the two groups of major blunt injury patients with head injury who received early VATS within 4 days and those whose treatment was delayed for more than 4 days are compared.

## Materials and methods

### Setting and patients

This observational study was conducted in a level I trauma medical center in Southern Taiwan with 1300 beds serving about 1200 trauma visits per month. During the study period, all patients admitted from our emergency department (ED) with major trauma with both blunt head and thoracic injuries were included. All patients’ data included demographics, the mechanism of injury, the number of ribs fractured, associated injuries, Injury Severity Score (ISS), Glasgow Coma Scale (GCS) at the emergency department and discharge, concomitant chest injuries, pulmonary contusion scores, postoperative complications, acute respiratory failure, number of ventilator days, length of stay (LOS) in the intensive care unit (ICU), and hospital LOS were prospectively collected with a standard form. A senior trauma surgeon reviewed all the data for accuracy. The ethics committee of the study hospital approved the study.

Patients older than 14 years of age admitted to the trauma unit in this hospital with blunt head and chest trauma were included. All trauma patients received computed tomography (CT) of the brain and chest, and those who had hemothorax or pneumothorax were treated with a 32F straight thoracostomy tube during initial evaluation at the ED with low-negative pressure (-15cmH_2_O). After detailed evaluations, all of these patients were admitted into the intensive care unit (ICU). Chest roentgenograms were routinely followed up daily. The chest tubes were adjusted when infiltration of routine chest x-ray is found. Bronchoscopy for sputum suction was also applied when atelectasis of lung was suspected. All of these patients received a brain CT and chest CT again at 24 to 48 h after trauma to evaluate the condition of intracranial hemorrhage and the volume of pleural collection [12]. Patients whose secondary chest CT showed a retained pleural collection persisting at more than 300 ml were considered for VATS. The retained hemothorax is calculated by formula *V* (in mL) = *d*^2^ × *X* × *L*, where *d* is the greatest depth of hemothorax from the chest wall to the lung on any CT image in centimeters, and *L* is the craniocaudal length in centimeters multiplied by the number of slices *X* centimeter thickness of CT cuts [13]. The thoracic surgeons were in charge of the VATS operations with the same criteria and surgical techniques used on every patient.

In this study, moderate to severe injuries to the abdomen and limbs with an Abbreviated Injury Score (AIS) over three were excluded. Patients with severe head injury with a mass effect for which an emergent craniotomy should be performed immediately were also excluded. Patients who were hemodynamically unstable, had more than 1500 mL of blood on placement of the initial tube thoracostomy, or had ongoing blood loss of more than 250 mL/h and had received an emergency thoracotomy were not included. Patients with blunt aortic injury were also excluded, as were patients with severe medical disease including chronic heart failure, end-stage renal disease, severe liver cirrhosis, and chronic obstructive pulmonary disease.

Time periods from trauma to VATS performance were variable in these patients. According to study of Yokobori et al. [4], these procedures are usually delayed, being performed more than 4 days after trauma for brain autoregulation recovery. In this study, some patients received VATS within 4 days after injury. For example, if a patient had a limb fracture needing fixation or a huge crush wound requiring debridement within short time after trauma, VATS would be arranged with these associate surgeries at the same time. Therefore, patients were divided into two groups based on the day of VATS performance: Group 1, within 4 days; Group 2, 5 or more days.

In this study, all the patients with head injury were observed without surgical intervention and received VATS due to retained hemothorax. The VATS was performed in the operating room under general anesthesia. Routine one dose of prophylactic antibiotics was given at the beginning of VATS. Evacuations of retained pleural collections were performed via VATS procedures. The pleural effusions were sent for microbial cultures during VATS in both groups. Finally, each patient was transferred to trauma ICU for further postoperative care. After either procedure, the thoracostomy tubes were connected to suction with low-negative pressure (-15cmH_2_O) and were removed at the discretion of the thoracic surgeons when drainage was less than 100 mL/24 h and no air leaks were present [14].

Post-trauma infection included positive microbial cultures of sputum, pleural effusion, and blood. Postoperative outcomes were in-hospital mortality, length of ventilator use, coma scale, and ICU and hospital LOS.

### Statistical analysis

An initial descriptive analysis was performed for every variable, determining frequencies and averages in the two groups. Numerical variables were presented as mean ± SD. Chi-square or Fisher’s test was used to evaluate categorical or proportional variables between the groups. Continuous variables between the groups were compared by analysis of variance. Statistical significant is considered as *p* < 0.05. All data were analyzed using the SPSS 18.0 statistical software. The power of ICU length of stay was 0.84, with sample size 61, Type I error rate (*α*) 5%, and sampling ratio = 1.

## Results

From May 2005 to February 2012, a total of 2023 patients with chest injuries were admitted to our hospital. Sixty-one patients had moderate to severe head injury with intracranial hemorrhage without indication of emergent craniotomy. They also had blunt chest injuries with complicated hemothorax or pneumothorax or both. All of these patients received thoracoscopic drainage because of clotted post-traumatic hemothorax after tube thoracostomies at the ED. Table 1 displays these patients’ characteristics. There were 45 men and 16 women, their ages ranging from 16 to 87 years (mean ± SD, 51.61 ± 18.82 years). The injury mechanisms were motorcycle-related injury in 46 patients, car driver or passenger injury in four patients, fall-related injuries in four patients, and bicycle rider or pedestrian injuries in seven patients. Their mean Injury Severity Scores (ISS) were 25.46 ± 6.00.

**Table 1:** Demographic analysis of patients treated with VATS for blunt trauma (*n* = 61)

Mean age ± SD (year)	51.61 ± 18.82
% Males	73.8%
Median ISS	25.46 ± 6.00
Mechanism of injury	
Motorcyclist or passenger	46
Car driver or passenger	4
Fall	4
Bicycle rider or pedestrian	7
Mortality (none related to VATS)	4.9% (3/61)
Associated injuries	
% Abdominal injury	34.4%(21/61)
% Extremity injury	75.4%(46/61)
Anatomic injury score of thoracic injury	3.23 ± 0.53
Anatomic injury score of head injury	3.25 ± 0.51
Pulmonary contusion score	6.44 ± 1.8
GCS at ED	11.74 ± 3.26
GCS at discharge	14.10 ± 2.78
Time from trauma to VATS (mean ± SD)	5.78 ± 3.22
ICU LOS (days)	13.38 ± 16.15
In-hospital LOS (days)	28.00 ± 19.66

*ISS* Injury Severity Score, *VATS* video-assisted thoracoscopy, *GCS* Glasgow Coma Scale, *ED* emergency department, *ICU* intensive care unit, *LOS* length of stay

The AIS of the patients’ thoracic region was 3.23 ± 0.53. The AIS of the head was 3.25 ± 0.51. Fifty-two patients had subarachnoid hemorrhage with intracerebral hemorrhage, six patients had subdural hemorrhage, and three patients had small epidural hemorrhage. There were no surgical indications for head injuries in these patients. The Glasgow Coma Scale (GCS) at initial assessment was 11.74 ± 3.26. When patients were discharged, the mean GCS returned to 14.10 ± 2.78 (paired *t* test, *p* < 0.05, excluding the expired patients). Patient mortality was 4.9% (3/61). The pulmonary contusion scores were calculated after an initial chest CT was obtained. The mean values were 6.44 ± 1.88. Thirty-six patients (36/61, 59.0%) had acute respiratory failure that needed endotracheal tube intubation with a ventilator for respiratory support within 24 h after trauma. The mean time between the trauma and the performance of the VATS was 6 days (5.77 ± 3.22). The mean (± SD) of ICU and hospital LOSs were 13.38 (±16.15) and 28.00 (±19.66) days.

In this study, all patients had tube thoracostomy before VATS. No VATS procedure was converted to thoracotomy during the surgical intervention. In order to realize the benefits of early VATS drainage for retained hemothorax in patients with combined head injuries, patients were divided into two groups according to the time from trauma to VATS performance. Group 1 patients received VATS within 4 days (mean 3.12 ± 0.49 days). Group 2 patients received surgery after more than 4 days (mean 8.35 ± 2.56 days). Patients in both groups received once VATS during their in-hospital course. Table 2 displays the results of the basic demographic comparisons between the two groups. The basic demographics, including age, gender, mechanism of injury, number of ribs fractured, pulmonary contusion scores, and AIS of head and chest and abdomen, showed no statistical differences between the groups. The rate of acute respiratory failure was not significantly different between the two groups (56.7 vs. 61.3%, *p* = 0.714). Therefore, the two groups of patients were statistically comparable and analogous.

**Table 2:** Comparison of basic demographics between patient groups

	Group 1 (*n* = 30)	Group 2 (*n* = 31)	p
Age (mean ± SD)	48.00 ± 16.49	55.06 ± 20.49	0.146
Gender (male)	21 (70.4%)	24 (77.4%)	0.510
ISS	25.47 ± 5.61	25.45 ± 6.45	0.992
Mechanism of injury			0.988
Motorcycle	23	23	
Car	2	2	
Fall	2	2	
Walk or bicycle	3	4	
Number of ribs fractured	5.73 ± 3.86	6.00 ± 3.34	0.774
Pulmonary contusion scores	6.50 ± 1.83	6.39 ± 1.96	0.817
Thoracic AIS	3.13 ± 0.43	3.32 ± 0.60	0.163
Head AIS	3.20 ± 0.48	3.29 ± 0.53	0.489
GCS at ED	11.67 ± 3.69	11.81 ± 2.86	0.869
GCS at discharge	14.10 ± 2.52	14.10 ± 3.05	0.869
Acute respiratory failure in 24 h	17 (56.7%)	19 (61.3%)	0.714

*ISS* Injury Severity Score, *AIS* anatomic injury score

The AIS of limbs was higher in Group 1, with statistical significance (1.87 ± 1.23 vs. 1.23 ± 0.92, *p* = 0.012). The ISS of Group 2 was slightly lower than that of Group 1, but there was no statistical clinical significance between the two groups (25.47 ± 5.61, and 25.45 ± 6.45, *p* = 0.992, respectively). Positive enterobacteria cultures from sputum in the two groups also showed no significant difference (13.3 vs. 9.7%, *p* = 0.654).

Table 3 shows the outcomes between the two groups. The ICU LOS and hospital LOS were both longer in Group 2 (*p* < 0.05, respectively). The total periods of ventilator use days were also longer in Group 2 (*p* = 0.016). The length of post-VATS ventilator use was longer in Group 2 (5.00 ± 7.39 and 11.33 ± 18.77, *p* = 0.079, respectively). The time interval of chest tube usage after VATS showed no statistical difference between the two groups (9.00 ± 4.59 and 9.52 ± 10.36, *p* = 0.802). The total time periods of chest tube insertion were shorter in Group 1 than in Group 2 (11.7 ± 5.37 and 16.38 ± 10.76, *p* = 0.036). Rate of pleural infections was increased in Group 2 (6.7 vs. 29.0%, *p* = 0.043).

**Table 3:** Comparison of clinical outcomes between patient groups

	Group 1 (*n* = 30)	Group 2 (*n* = 31)	*p*
ICU LOS	8.97 ± 6.49	17.65 ± 21.03	0.035
Hospital LOS	20.63 ± 9.08	35.13 ± 24.21	0.003
Total ventilator days	6.77 ± 7.31	18.55 ± 24.94	0.016
Post-VATS ventilator days	5.00 ± 7.39	11.33 ± 18.77	0.079
Chest tube use days	11.70 ± 5.37	16.38 ± 10.77	0.037
Post-VATS chest tube use days	9.00 ± 4.59	9.51 ± 10.36	0.802
Positive microbial cultures			
Sputum	10 (33.3%)	16 (51.6%)	0.198
Pleural effusion	2 (6.7%)	9 (29.0%)	0.043

## Discussion

Head and chest injuries frequently occur together in blunt trauma [15, 16]. In multiple injuries, maintaining stable neurological status is the first priority. Thus, the timing of urgent surgical interventions will be delayed when a patient has a head injury. However, many recent reports suggest that early evacuation of the pleural collection within 4–5 days after trauma could achieve better clinical outcomes [10, 15, 16]. In this study, early surgical intervention in chest injury really could obtain a better outcome in patients with stable head injury.

Acute respiratory failure is prone to induce post-traumatic infections [8, 14]. These two complications are the main reasons for prolonging the in-hospital course. These complications occur with high frequency in the case of combined head and chest injuries. In this study, half of the patients (36/61, 59.0%) required emergent endotracheal tube intubation because of a change of consciousness or a rapid decrease in oxygen saturation. The rate of acute respiratory failure was equal in the two groups. Many studies have proofed that shorting the periods of ventilator dependent could also decrease the rate of pneumonia. In this study, there were 26 patients who had post-traumatic pneumonia that were derived from aspiration pneumonia and nosocomial pneumonia. The rate of enterobacterial cultures from sputum was similar in each group. It means that rate of aspiratory pneumonia was also equal in each group. In Group 1, early VATS could re-expand the lung parenchyma earlier that could restore the lung function more rapidly. By decreasing the ventilator dependent periods, the rate of nosocomial pneumonia may also be decreased indirectly.

The rate of positive microbial cultures from pleural effusion obtained during VATS was much lower in Group 1 with statistical difference. It means that early VATS in Group 1 could remove retained hemothorax more early to prevent further pleural infections. That is, the retained hemothorax had been infected with bacteria in most Group 2 patients. The antibiotics treatment course in these patients will be lengthened. Due to the early control of infection in Group 1, the antibiotic requirements and cost were both decreased. This also made it easier to gradually discontinue the ventilator use. These advantageous effects played an important role in shortening the whole hospital course.

The basic demographic data were the same in each group in this study. The greatest difference between these two groups was found in limb injuries. All patients in Group 1 had limb injuries, such as open fractures or neurovascular injuries, requiring early damage control intervention. During these surgeries, VATS was performed simultaneously for retained pleural collections. In most cases, the surgical time of VATS was less than 30 min, without any increase in the total surgical periods.

In this study, time periods of ventilator and chest tube use after VATS were collected to evaluate the successful rates of VATS between the two groups. The total time periods of ventilator and chest tube usage were both longer in Group 2, but the postoperative time periods were the same in the two groups, signifying that VATS was performed with same efficacy in both groups. The longer total time periods in Group 2 resulted from the longer waiting time for monitoring the neurological condition. In this study, the VATS could be performed safely within 4 days or less. All patients’ GCS scores were improved when they were discharged, and the GCS at discharge showed no difference between the two groups, signifying that the early surgical procedure did not influence these patients’ brain condition. The routine waiting time of at least 4 days in head injury patients without indication of emergency craniotomy may be unnecessary.

Due to advancement of ICU care, the mortality of severe and multiple trauma patients is much lower than before [2, 16, 17]. In this study, only three patients expired: the mortality rate was 4.9% (3/61). One patient in Group 1 had progressively scattered intracranial hemorrhage and unfortunately expired due to central failure. The other two in Group 2 had severe pneumonia-induced septic shock that induced multiple-organ failure. The other patients all recovered after comprehensive care in the ICU. Therefore, under detail monitoring and treatments, VATS could be performed earlier in patients whose brain condition is relatively stable.

This is the first study to observe the early VATS intervention in chest trauma with brain injury. However, this study has several limitations. First, the case numbers in this study were low. Only 61 patients who qualified for the inclusion criteria during the study period were enrolled. It was difficult to find qualifying patients because only 10% of chest blunt trauma cases exhibit retained pleural collections, and also because cases of severe head injuries with emergent craniotomy were excluded. However, the preoperative status and demographic matching of the two groups decrease the bias, and the power of this study is 0.84; therefore, fewer case numbers may not influence the accuracy of the statistical analysis. Second, although this study was based on prospective data collection, some of the parameters were collected from chart reviews. Errors may have occurred during recording. To prevent this bias, a senior trauma surgeon supervised a rechecking of the exact data. Third, many factors could influence the duration of acute respiratory failure. Severe abdominal injury could lengthen the in-hospital stay. Chronic medical problems such as chronic obstructive pulmonary disease, liver cirrhosis and chronic heart failure could also increase morbidity and mortality. All of these biases were excluded from enrollment in this study. In addition, aspiration pneumonia is another complication after trauma, especially for head injury with unconscious status [18]. If the rate of positive culture from enterobacteria was the same in these two groups, this bias would therefore also be decreased. Finally in recent studies, thrombolytic agents provide another choice for treating retained hemothorax [19]. Patients who are not candidates for VATS, intra-pleural irrigation with thrombolytic agents may also be considered.

## Conclusion

In patients with a combination of chest and head injuries without indication of emergency craniotomy, earlier VATS could be performed safely to obtain better clinical outcomes.
